# Heat transfer enhancement using CO_2_ in a natural circulation loop

**DOI:** 10.1038/s41598-020-58432-6

**Published:** 2020-01-30

**Authors:** L. R. Thippeswamy, Ajay Kumar Yadav

**Affiliations:** Department of Mechanical Engineering, National Institute of Technology Karnataka, Surathkal, Mangalore, 575025 India

**Keywords:** Mechanical engineering, Fluid dynamics

## Abstract

The natural circulation loop (NCL) is a highly reliable and noise-free heat transfer device due to the absence of moving components. Working fluid used in the natural circulation loop plays an important role in enhancing the heat transfer capability of the loop. This experimental study investigates the subcritical and supercritical heat transfer performance of a natural circulation loop (NCL) with CO_2_ as the working fluid. Operating pressures and temperatures are varied in such a way that the loop fluid should remain in the specified state (subcooled liquid, two-phase, superheated vapor, supercritical). Water and methanol are used as external fluids in cold and hot heat exchangers for temperatures above zero and below zero (in °C) respectively, depending on operating temperature. For loop fluids, the performance of CO_2_ is compared with water for above zero and with brine solution for the subzero case. Further, the impact of loop operating pressure (35–90 bar) on the performance of the system is also studied. For hot heat exchanger inlet temperature (5 to 70 °C) and cold heat exchanger inlet temperature (−18 to 32 °C), it was observed that the maximum heat transfer rates in the case of subcritical vapor, subcritical liquid, two-phase and supercritical CO_2_ based systems are 400%, 500%, 900%, and 800% higher than the water/brine-based system respectively.

## Introduction

The heat transfer loops (secondary loops) are classified as forced circulation loop (FCL) and the natural circulation loop (NCL). Forced circulation loop is an active system which requires pump or compressor to drive the fluid flow, whereas natural circulation loop (NCL) is a simple system in which fluid flow takes place due to the density gradient caused by an imposed temperature difference.

In an NCL, the heat sink is situated at a higher elevation than the heat source. This establishes a density gradient in the system, due to which, lighter (warmer) fluid rises up and heavier (cooler) fluid moves down. Hence thermal energy can be transported from a high temperature source to a low temperature sink without direct contact with each other and also without using any prime mover.

NCL is preferred over forced convection loop where safety is the foremost concern. It also provides noise free and maintenance free operations. NCL is a promising option in many engineering applications such as nuclear reactors^1^, chemical extraction^2,3^ electronic cooling system^4^, solar heaters^5–10^, geothermal applications^11,12^, cryogenic refrigeration systems^13^, turbine blade cooling^14^, thermosyphon reboilers^15,16^, and refrigeration and air conditioning^17^, etc. Compared to forced convection systems, heat transfer rates in natural convection systems are on the lower side, and enhancement of the same is a challenging task. Researchers are trying different ways for the improvement of heat transfer rate such as by using various working fluids/nanofluids. Misale *et al*.^18^ and Nayak *et al*.^19^ experimentally reported a 10–13% enhancement in heat transfer rate with nano-fluid (Al_2_O_3_ + water) compared to water based NCL.

The selection of working fluids for NCL is typically carried out based on some favorable thermophysical properties. Commonly used working fluids can be divided into aqueous and non-aqueous category. Aqueous solutions are generally either salt based or alcohol based products. These are having one or more non-favorable effects like corrosiveness, toxicity, high pH value, etc. Non-aqueous solutions are commercially available chemicals.

In recent years, CO_2_ has gained popularity as a loop fluid in NCL due to its excellent thermophysical properties and environment benignity (no ozone depletion potential and negligible global warming potential) and has been employed for various applications such as solar thermal collector^20^, heat pump^21^, geothermal system^22^, etc. Suitability of CO_2_ as a loop fluid has been studied by Kiran Kumar *et al*.^23^ for NCL, and by Yadav *et al*.^24^ for forced circulation loop.

Any fluids operating near-critical region, show very good heat transfer and fluid flow characteristics due to its favorable thermophysical properties. Carbon dioxide has an advantage of low critical temperature (~ 31 °C) and quite reasonable critical pressure (73.7 bar).

Swapnalee *et al*.^25^ carried out experimental investigations to study the static instability of supercritical CO_2_ and water-based NCLs with heater as a heat source. Kiran *et al*.^26^ conducted experiments and studied the heat transfer behavior of NCL using subcritical CO_2_ with limited temperature and pressure range.

Although, the availability of experimental studies is very scant due to the risk involved in handling high operating pressure of CO_2_, a quite good number of numerical studies on the heat transfer behavior of CO_2_ based NCLs are available in open literature^27–29^.

Kiran Kumar *et al*.^27^ carried out a numerical study on steady-state analysis of single-phase rectangular NCL with parallel flow, tube-in-tube type heat exchangers. Yadav *et al*.^28^ carried out transient analysis of carbon dioxide-based natural circulation loop (NCL) with end heat exchangers. Basu *et al*.^29^ carried out, aims at the development of a theoretical model to simulate the steady-state performance of a rectangular single-phase natural circulation loop and to investigate the role of different geometric parameters on the system behavior. Yadav *et al*.^30^ carried out three dimensional CFD study and claimed ~700% higher heat transfer rate in the case of subcritical liquid as well as supercritical CO_2_ compared to water. Two-dimensional analysis at 90 bar for various heat source temperatures reported the instabilities associated with supercritical flow^31,32^.

Ample numerical studies^27–29^ on CO_2_ based NCL with different configurations are available. However, very few experimental studies are reported in the literature on account of the risk involved in handling CO_2_ at higher operating pressure. As in most of the engineering studies pertaining to practical relevance, experimental studies form the benchmark. The experimental studies on NCLs employing supercritical/subcritical CO_2_ with end heat exchangers over a wide range of temperatures covering the subzero temperature are limited. To fill in that critical void, this experimental study presents an investigation on the heat transfer behavior of subcritical/supercritical CO_2_ based NCL with end heat exchangers for the wide applications ranging from subzero (−18 °C) to above zero (70 °C) temperatures. The study also includes the heat transfer phenomenon in a single phase (liquid and vapor) and two-phase CO_2_ based NCL. Further heat transfer rates of water (for above zero temperature) and brine solution (for subzero temperature) in NCL are compared.

### The Experimental details

**Figure 1:**
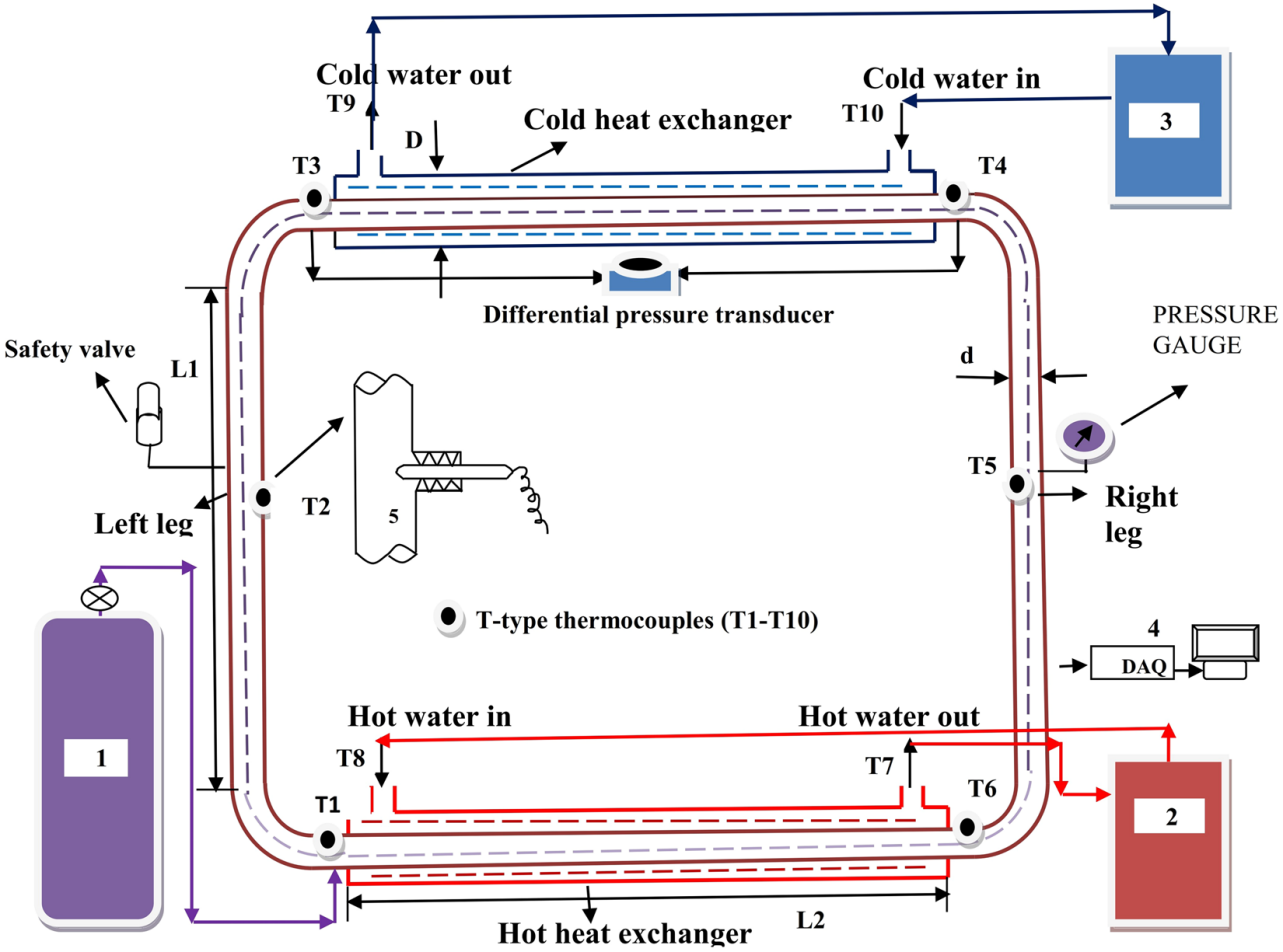
Schematic of the NCL with end heat exchangers. (1) CO_2_ reservoir cylinder, (2) Thermostatic bath for HHX, (3) Thermostatic bath for CHX (4) Data acquisition system, (5) Enlarge portion of inside thermocouple arrangement (Nut and ferrule).

A complete representation of the test facility is in Fig. 1. The test facility comprises of a CO_2_ reservoir, tube-in-tube heat exchangers (hot and cold) with vertical tubes (riser and downcomer).

T-type thermocouples of appropriate length are connected to measure temperature of the loop fluid (CO_2_/water/brine solution) and external fluid (water/methanol) that flows inside the inner tube and the annulus, respectively as depicted in Fig. 1.

**Figure 2:**
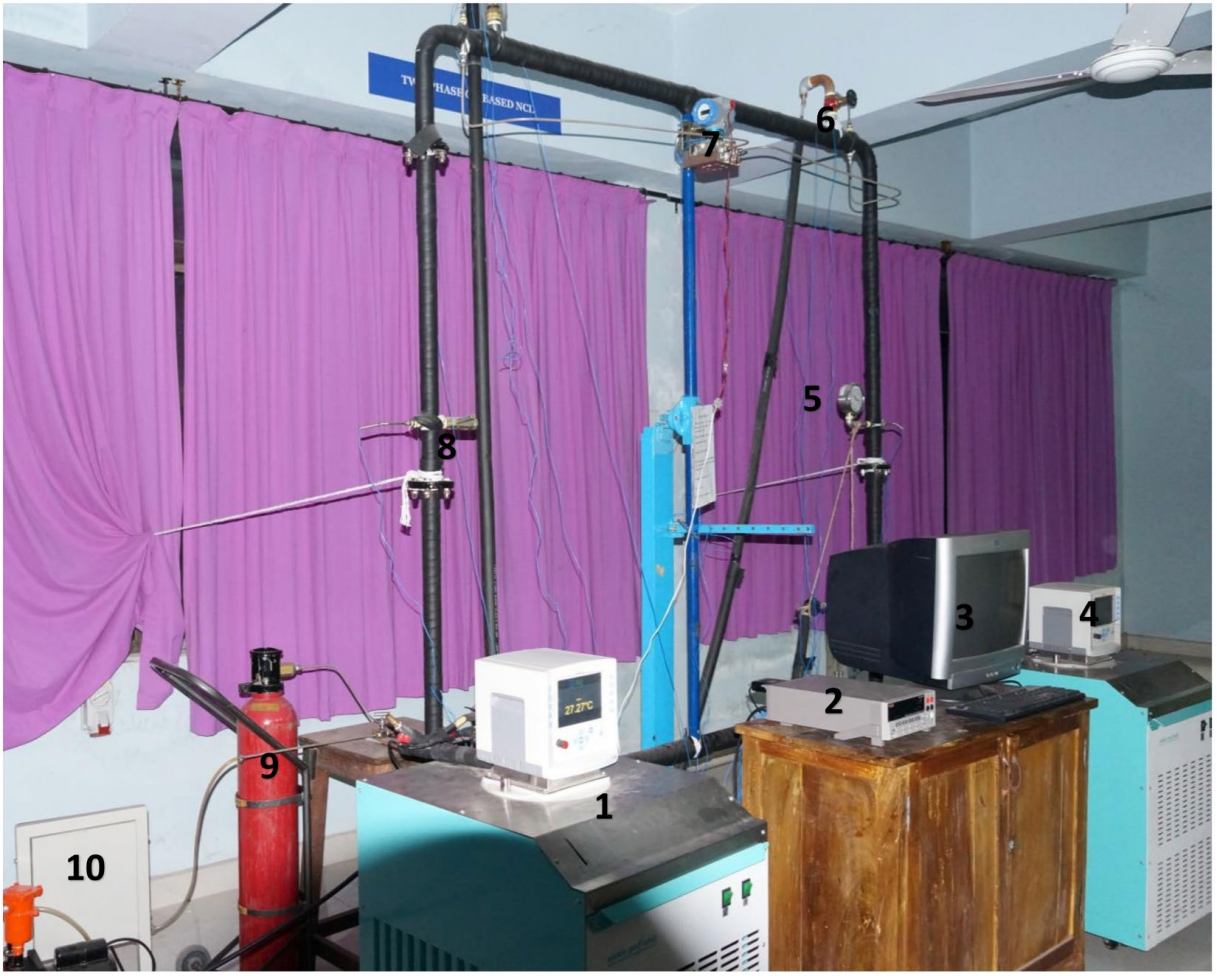
Experimental setup. (1) Thermostatic bath- 1(HHX), (2) DAQ, (3) Computer to read DAQ Data, (4) Thermostatic bath −2(CHX), (5) Pressure Gauge, (6) Rotameter, (7) Differential Pressure Transducer, (8) Safety Valve, (9) CO_2_ cylinder, (10) Vaccum pump.

The photographic view of the employed facility is shown in Fig. 2. Natural circulation loop of 2 × 2 m is made up of stainless steel (SS-316) having outer diameter 32 mm, inner diameter 26 mm, thickness 3 mm and it withstands pressure up to 250 bar. To control heat transfer from loop to ambient, the entire loop is insulated with asbestos rope and foam tape insulating material of 3 mm thick each. The heat exchangers of length of 1600 mm, outer diameter 51 mm, and having thickness 3 mm.

Two thermostatic baths (Thermo scientific PC200) having a heating/cooling capacity of 2 kW supplies external fluid (water/methanol) at fixed temperature to the heat exchangers. Mass flow rate of external fluids is measured using two calibrated rotameters (2–20 LPM range) with valve arrangement, connected separately to HHX and CHX.

A bourdon pressure gauge range of 0–150 bar is connected to measure loop line pressure at the center of the right leg. Six T-type thermocouples are used to monitor the temperature of CO_2_ at various locations along the loop, thermocouples are connected in direct connection with the internal loop fluid CO_2_ as shown in Fig. 1 of enlarged portion of nut and ferrule arrangement. A data acquisition system (DAQ, Keighley - Model 2700) is employed to record various temperatures of the loop. The geometrical specifications of the test rig are specified in Table 1. The operating variables and its operating range is presented in Table 2 for the entire experiment.

**Table 1:** Geometrical parameters of the experimental setup.

Loop details	Size (mm)
Outer diameter of the loop pipe (d)	32
Internal diameter of the loop pipe	26
Thickness of the loop pipe	3
Length of left leg or right leg loop(L1)	1800
Length of the bend of the loop (outer)	157
Length of the bend of the loop (inner)	122.5
Distance from heat exchanger up to bend of the loop	100
**Heat exchanger details**	
Outer Diameter of the heat exchanger (D)	51
Thickness of the heat exchanger outer wall	4
Length of heat exchanger (L2)	1600
Annulus distance (radial)	5.5

**Table 2:** Range of operating parameters considered during study.

Parameters	Range	Error range (%)
Hot water inlet temperature (*T*_*H*_)	−10–70 °C	±0.05
Coldwater inlet temperature (*T*_*C*_)	−18–32 °C	±0.05
System pressure	35–90 bar	±2.5
External fluid mass flow rate (m)	5 LPM	±5.0

### Methodology

The cold and hot heat exchangers are tested for leakages up to 10 bar pressure, and the loop is tested for leaks at 150 bar. Later, entire natural circulation loop is evacuated, and required amount of CO_2_ is charged to the loop from CO_2_ cylinder. Charging of CO_2_ is stopped once the loop fluid pressure reaches required operating condition. External fluid is made to flow inside annular tube of both heat exchangers at specified mass flow rate and temperatures. When external fluid starts flowing, loop temperature starts varying with small variation in loop pressure. To maintain specified operating pressure, CO_2_ is transferred to/from the cylinder which is kept at operating pressure. This practice continues until the loop reaches steady state. The loop is said to be reached steady state if the transient variation in all temperatures and pressures are less than 0.5%.

At specified operating pressure, the state of CO_2_ is confirmed by monitoring the temperature at all locations of the loop (single-phase, two-phase or supercritical phase). Once the complete system reaches a steady-state, results are recorded. To compare the results of CO_2_ as loop fluid, a brine solution is used as loop fluid for lower temperature applications, whereas water is used for above zero temperature applications. Methanol is used as an external fluid for lower temperature (below 0 °C) applications and water as an external fluid for higher temperature (above 0 °C) applications.

To ensure turbulent flow conditions for the external fluid, a mass flow rate of 0.083 kg/s (5 liters/min) is maintained in CHX as well as in HHX.

Heat transfer rate (Q) is calculated by

1 $${\rm{Q}}={\rm{m}}\times {{\rm{c}}}_{p-HHX}\times {\Delta {\rm{T}}}_{{\rm{HHX}}}={\rm{m}}\times {{\rm{c}}}_{p-CHX}\times {\Delta {\rm{T}}}_{{\rm{CHX}}}$$

where, m = mass flow rate of external fluid in kg/s

c_*p*–*HHX*_ = specific heat of HHX in J/kg-K

c_*p*–*CHX*_ = specific heat of CHX in J/kg-K

ΔT_HHX_ = HHX temperature difference between inlet and outlet

ΔT_CHX_ = CHX temperature difference between inlet and outlet

Average temperature is calculated by

2 $${T}_{avg}=\frac{{T}_{C}+{T}_{H}}{2}$$

where,*T*_*C*_ = CHX inlet temperature in °C

*T*_*H*_ = HHX inlet temperature in °C

## Results and Discussion

This experimental study covers wide applications ranging from −18 °C to 70 °C temperatures and operating pressure from 35 bar to 90 bar. Heat transfer rate, pressure drop and temperature distribution of single phase (supercritical, liquid and vapor) and two-phase CO_2_ based NCL compared with water/brine based natural circulation loop at same operating temperatures. The operating pressure for water and brine as loop fluid is kept at 1 atm pressure as the variation of thermophysical properties of water with operating pressure is insignificant (less than 1%), which in turn does not affect the heat transfer rate significantly^33^.

### Supercritical CO_2_ as loop fluid

**Figure 3:**
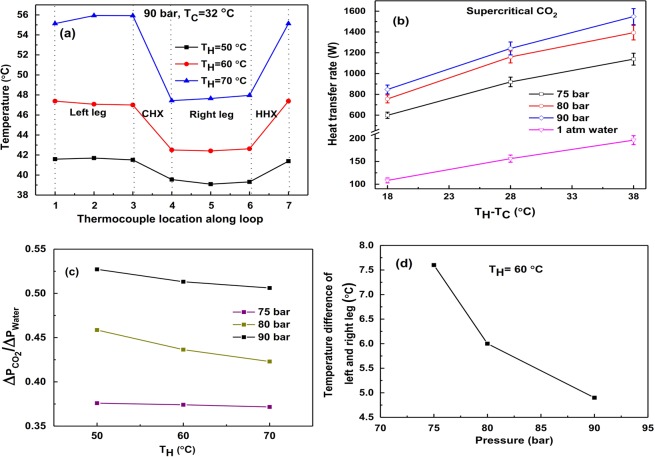
For Supercritical CO_2_: (**a**) Temperature at different points along the loop, (**b**) Variation of heat transfer rate for water and CO_2_ at different pressures, (**c**) Pressure drop comparison of water and CO_2_ at different pressures, (**d**) Temperature difference between left and right legs v/s operating pressure.

In CHX and HHX, water is the external fluid. For a fixed inlet temperature of water (just above the critical temperature of CO_2_ ~31.2 °C), HHX inlet temperature is varied from 40 °C to 70 °C in steps of 10 °C. Figure 3(a) shows the temperature variation throughout the loop at 90 bar. The temperature variation is also recorded for all operating pressures to make sure the loop fluid is in supercritical state throughout the loop.

The effect of CO_2_ pressure on the heat transfer rate and pressure drop is studied by varying it from 75 to 90 bar in a supercritical zone as shown in Fig. 3(b,c). Heat transfer rate is compared with widely used loop fluid i.e., water at atmospheric pressure (1 atm) under the same HHX and CHX temperatures. Figure 3(d) shows the effect of pressure on the loop fluid temperature difference between the left leg center and right leg center. Results clearly show that as pressure increases, temperature difference decreases, which occurs due to an increase in specific heat at higher pressure at particular average operating temperature (T_avg_ = 46 °C, T_H_ = 60 °C) as shown in Table 3. At higher temperatures, decrease in viscosity leads to lower pressure drop in the loop (Fig. 3(c)). Uncertainty (error) analysis has been carried out (shown after results and discussion part), and errors are incorporated in heat transfer calculation for all the cases.

**Table 3:** Comparison of the properties of supercritical CO_2_ at different pressures with water at atmospheric pressure for different operating temperatures^33^.

Pressure of CO_2_ (bar)	Avg. Temperature, T_avg_(°C)	Density ratio, ρ_CO2_/ρ_Water_	Specific heat ratio, C_p_CO2_/ C_p_Water_	Thermal conductivity ratio, k_CO2_/k_Water_	Viscosity ratio, μ_CO2_/μ_Water_	Ratio of volumetric coefficient, β_CO2_/β_Water_
75	41	0.23	0.75	0.05	0.03	56.68
	46	0.21	0.59	0.05	0.03	37.89
	51	0.19	0.50	0.05	0.04	28.21
80	41	0.27	1.05	0.06	0.03	83.50
	46	0.24	0.72	0.05	0.04	48.29
	51	0.22	0.58	0.05	0.04	33.70
90	41	0.44	2.89	0.11	0.05	240.54
	46	0.33	1.27	0.07	0.04	91.65
	51	0.28	0.83	0.06	0.04	51.69

The effect of operating pressure on the heat transfer rate at different HHX inlet temperatures (T_H_) for a fixed T_C_ is depicted in Fig. 3(b). The heat transfer rate is found to be maximum for the operating pressure of 90 bar. Average operating temperature (~loop fluid temperature) of 41 °C (obtained in this case) is near to the pseudo critical point (40.2 °C) of CO_2_ at 90 bar, which leads to a maximum heat transfer rate at this pressure because of a very high volumetric expansion coefficient of CO_2_ compared to water (~240 times). Experiments are also carried out for average operating temperatures of 46 °C and 51 °C.

In this case, the maximum heat transfer rate of CO_2_ based NCL yields ~8 times (800%) higher than water-based NCL as shown in Fig. 3(b). At higher HHX inlet temperature, buoyancy effect predominates increasing the heat transfer rate.

### Subcritical vapor CO_2_ as loop fluid

**Figure 4:**
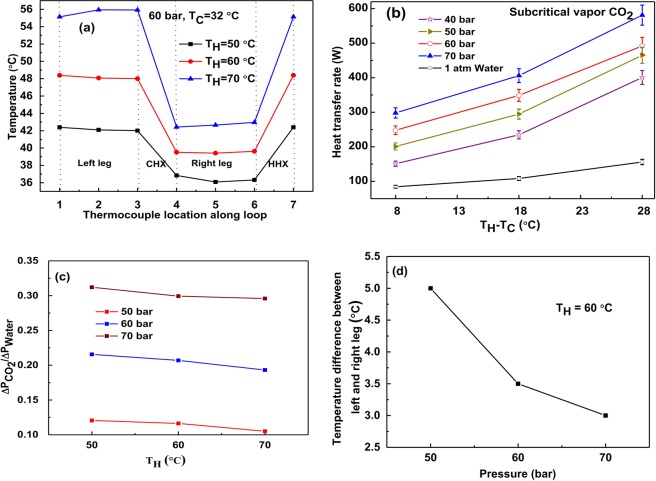
For Subcritical CO_2_ vapor: (**a**) Temperature at different points along the loop, (**b**) Variation of heat transfer rate for water and CO_2_ at different pressures, (**c**) Pressure drop comparison of water and CO_2_ at different pressures, (**d**) Temperature difference of left and right legs v/s pressure.

With water as the external fluid in both CHX and HHX, for a fixed inlet temperature in CHX (=32 °C), the inlet temperature in HHX is varied from 40 °C to 70 °C for incremental values of 10 °C. For varying operating pressures of CO_2_ (40 to 70 bar), data is collected. Figure 4(a–d) shows the temperature variation along the loop, impact of operating pressure on the heat transfer rate, pressure drop v/s operating pressure, and temperature difference between left and right legs v/s operating pressure for subcritical vapor case. Figure 4(a) shows the temperature variation throughout the loop at 60 bar. It is observed that with increase in hot fluid inlet temperature heat transfer rate increases due to an increase in the temperature gradient between CO_2_ and water in HHX. With the increase in system pressure, the heat transfer rate also increases. In this case, the maximum heat transfer rate of CO_2_ based NCL yields ~4 times (400%) higher than water-based NCL (1 atm) for the same operating temperatures as shown in Fig. 4(b). The difference in pressure drop is found to be insignificant for the operating pressure between 40–70 bar as shown in Fig. 4(c) which occurs due to constant viscosity ratio (shown in Table 4). Results show the decrease in temperature difference between left and right legs as operating pressure increases as depicted in Fig. 4(d).

**Table 4:** Comparison of the properties of subcritical vapor CO_2_ at different pressures with water at atmospheric pressure for different operating temperatures^33^.

Pressure of CO_2_ (bar)	Avg. Temperature, T_avg_(°C)	Density ratio, ρ_CO2_/ρ_Water_	Specific heat ratio, C_p_CO2_/ C_p_Water_	Thermal conductivity ratio, k_CO2_/k_Water_	Viscosity ratio, μ_CO2_/μ_Water_	Ratio of volumetric coefficient, β_CO2_/β_Water_
40	41	0.08	0.30	0.03	0.03	16.11
	46	0.08	0.29	0.03	0.03	13.83
	51	0.07	0.28	0.03	0.03	12.08
50	41	0.11	0.35	0.04	0.03	20.56
	46	0.11	0.33	0.04	0.03	17.17
	51	0.11	0.32	0.04	0.03	14.67
60	41	0.15	0.43	0.04	0.03	27.92
	46	0.14	0.40	0.04	0.03	22.27
	51	0.14	0.37	0.04	0.03	18.39
70	41	0.20	0.59	0.05	0.03	42.45
	46	0.18	0.50	0.05	0.03	30.91
	51	0.17	0.45	0.04	0.03	24.09

### Subcritical liquid CO_2_ as loop fluid

**Figure 5:**
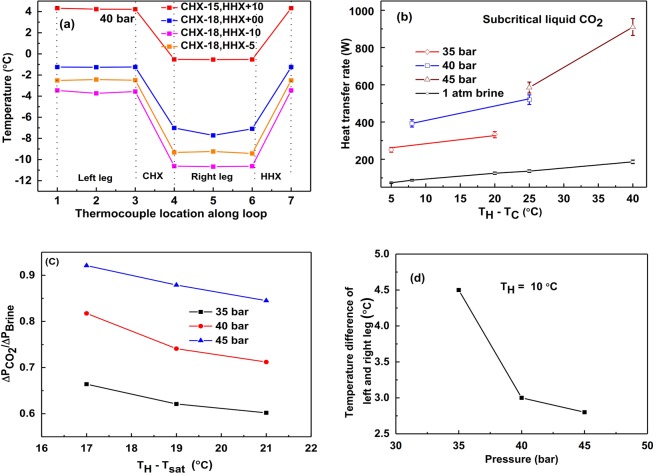
For subcritical liquid CO_2_: (**a**) Temperature at different points along the loop, (**b**) Variation of heat transfer rate for brine and CO_2_ at different pressures, (**c**) Pressure drop comparison of brine and CO_2_ at different pressures, (**d**) Temperature difference of left and right legs v/s pressure.

This experimental study is mainly focused on low temperature (below 0 °C) applications such as refrigerators, solar water heater for cold weather, etc. In CHX and HHX, methanol is used as the external fluid as water becomes solid at sub-zero temperature. The inlet temperature of CHX is maintained constant and HHX temperature is varied. To compare the heat transfer rate of liquid CO_2_ based NCL, we conducted experiments using brine solution (a widely used fluid for sub-zero temperature) as loop fluid. Figure 5(a–d) shows the temperature variation along the loop, the heat transfer rate for different operating pressure, pressure drop v/s operating pressure, and temperature difference between left and right legs v/s operating pressure for subcritical liquid case. To ensure the liquid phase (of CO_2_) throughout the loop, temperatures at different locations are recorded as shown in Fig. 5(a). Since brine viscosity is higher than water, we will certainly get a lower heat transfer rate with brine. However, we achieved the maximum 500% higher heat transfer rate in this case of liquid CO_2_ compared to brine-based NCL as shown in Fig. 5(b). As explained earlier Fig. 5(c,d) shows similar trends of pressure drop and temperature difference for an increase in operating pressure respectively. Table 5. shows the comparison of the properties of subcritical liquid CO_2_ at different pressures with brine at atmospheric pressure for different operating temperatures, there is not much variation in viscosity ratio of CO_2_ and brine.

**Table 5:** Comparison of the properties of subcritical liquid CO_2_ at different pressures with brine at atmospheric pressure for different operating temperatures^33^.

Pressure of CO_2_ (bar)	Avg. Temperature, T_avg_(°C)	Density ratio, ρ_CO2_/ρ_brine_	Specific heat ratio, C_p_CO2_/C_p_ brine_	Thermal conductivity ratio, k_CO2_/k_brine_	Viscosity ratio, μ_CO2_/μ_brine_	Ratio of volumetric coefficient, β_CO2_/β_brine_
35	−16.5	0.89	0.64	0.25	0.004	16.19
	−14.5	0.89	0.64	0.25	0.004	16.52
40	−2.5	0.83	0.70	0.21	0.002	19.48
	−9	0.86	0.66	0.23	0.003	17.34
	−14	0.89	0.64	0.23	0.004	16.30
45	−2.5	0.84	0.69	0.21	0.003	18.86
	2.5	0.81	0.73	0.20	0.002	21.27

### Two-phase CO_2_ as loop fluid

**Figure 6:**
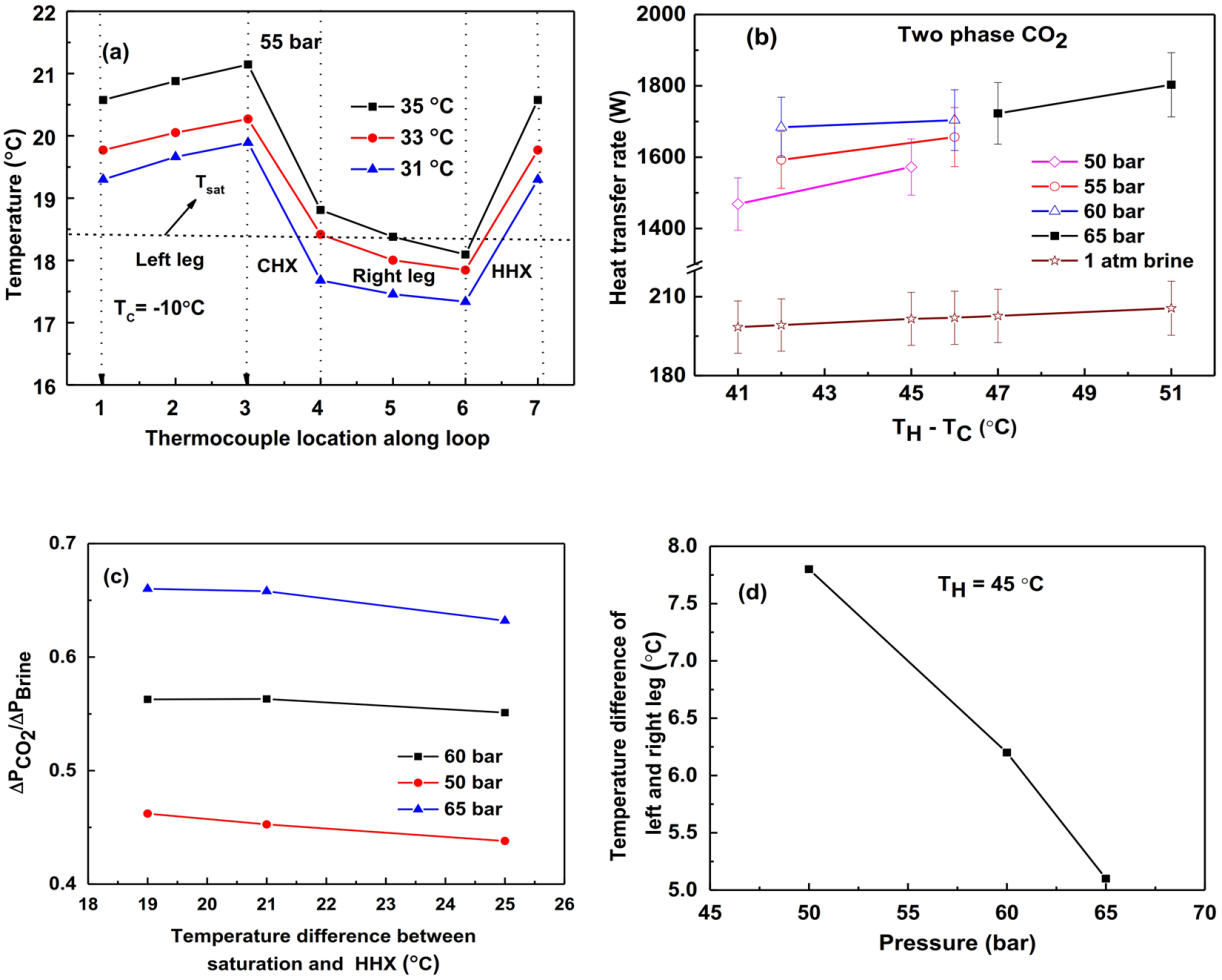
For two-phase CO_2_ condition (**a**) Temperature at different points along the loop, (**b**) Variation of heat transfer rate for brine and CO_2_ at different pressures, (**c**) Pressure drop comparison of brine and CO_2_ at different pressures, (**d**) Temperature difference of left and right legs v/s pressure.

In this study, methanol is employed as the external fluid in both CHX and HHX to achieve two-phase at lower temperatures (sub-zero temperature). The operating parameters considered to conduct the experiments are shown in Table 6. For different operating pressures of CO_2_, i.e., 50, 55, 60, and 65 bar, results are obtained. Similar to the liquid case, we carried out experiments using brine solution as loop fluid to compare the heat transfer rate of two-phase CO_2_ based NCL. Figure 6(a–d) shows the temperature variation along the loop, the heat transfer rate for different operating pressure, pressure drop v/s operating pressure, and temperature difference between left and right legs v/s operating pressures for two-phase CO_2_ case (liquid + vapor). In this case, achieving two-phase inside the loop maintained at high pressure is quite difficult. With the continuous record of temperatures at different locations in the loop, we achieved two-phase CO_2_ by comparing saturation temperature at a given pressure (shown in Fig. 6(a)).

**Table 6:** Operating parameters for two phase CO_2_.

Pressure (bar)	Saturation temperature (°C)	CHX Inlet temperature (°C)	HHX inlet temperature (°C)	Difference between saturation temperature and HHX inlet temperature (°C)
55	18.42	−10	35	21
			33	19
			31	17
60	22.13	−3	43	21
			41	19
			39	17
65	25.6	0	47	21
			45	19
			43	17

As the loop moves into the two-phase region, a large buoyancy effect gets generated causing an increase in the mass flow rate of CO_2_ and which in turn enhances the heat transfer coefficient. In this case, the maximum heat transfer rate of CO_2_ based NCL yields 9 times (900%) higher than the brine solution based NCL for the same operating temperatures as shown in Fig. 6(b). Figure 6(c) show the pressure drop variation at different operating pressures and different temperatures. It is interesting to see the effect of operating pressures on the temperature gradient in the left and right legs as shown in Fig. 6(d). As pressure decreases, the latent heat of vaporization increases which causes a decrease in temperature difference.

### Error analysis

Heat transfer rate, mass flow rate and temperature are the various performance parameters for functional dependency (specific heat of external fluid is considered to be constant), the relation is given as:

3 $${Q}_{HHX}=f(m,\,\Delta {T}_{HHX})$$

4 $${Q}_{CHX}=f(m,\,\Delta {T}_{CHX})$$

If *M* is a certain measuring parameter, its functional relationship with the independent variables as represented by *M* = *f*(*y*_1_, *y*_2_, *y*_3_, *y*_4_,…… + *y*_*n*_) then uncertainty in various parameter is given as:

5 $${u}_{R}={\left[{\left(\frac{\partial M}{\partial {y}_{1}}{u}_{1}\right)}^{2}+{\left(\frac{\partial M}{\partial {y}_{2}}{u}_{2}\right)}^{2}+{\left(\frac{\partial M}{\partial {y}_{3}}{u}_{3}\right)}^{2}+\ldots +{\left(\frac{\partial M}{\partial {y}_{n}}{u}_{1}\right)}^{2}\right]}^{1/2}$$

where *u*_1_, *u*_2_, *u*_3_, ………, *u*_*n*_ be the uncertainties in the independent variables.

With a rotameter of least count (0.2 LPM), minimum flow rate recorded is 5LPM.

Uncertainty associated with mass flow rate is

6 $$\frac{\Delta m}{m}=\frac{0.2}{5}=\pm \,0.04=\pm \,4.0 \% $$

Minimum operating temperature recorded is −18 °C and accuracy for T-type thermocouple is 0.25 °C.

Maximum uncertainty in temperature measurement is

7 $$\frac{\Delta T}{T}=\frac{0.25}{18}=\pm \,0.013=\pm \,1.3 \% $$

Heat transfer rate with considering uncertainty is calculated by

8 $$\begin{array}{c}\frac{\Delta Q}{Q}={\left[{\left(\frac{\Delta m}{m}\right)}^{2}+2{\left(\frac{\Delta T}{T}\right)}^{2}\right]}^{1/2}\\ \frac{\Delta Q}{Q}={\left[{\left(0.04\right)}^{2}+2{\left(0.013\right)}^{2}\right]}^{1/2}=0.0421=4.21 \% \end{array}$$

## Conclusion

Steady-state behavior of a CO_2_ based NCL is experimentally analyzed. Subcritical (liquid, vapor, and two-phase) and supercritical phases of the CO_2_ are studied for 35–90 bar and −18 to 70 °C. The heat transfer rate of CO_2_ based NCL is compared with widely used loop fluid i.e., water (for above 0 °C) and brine (for below 0 °C). Conclusions from the test results can be enumerated as follows:

- In the case of supercritical CO_2_ as loop fluid, the maximum increase in heat transfer rate is 800% higher compared to water as loop fluid.
- In the case of subcritical vapor CO_2_ as loop fluid, the maximum increase heat transfer rate is 400% higher compared to water as loop fluid.
- For low-temperature applications (below 0 °C), subcritical liquid CO_2_ yields a maximum 500% higher heat transfer rate compared to brine solution as loop fluid.
- Much higher heat transfer rate (maximum 900%) is obtained in the case of two-phase CO_2_ based NCL compared to brine-based NCL. This study is carried out for low-temperature applications (below 0 °C).

The present study will be useful in designing compact heat transfer devices for electronic cooling, refrigeration, and air conditioning, solar thermal collector, etc.

## Supplementary information

Heat transfer enhancement using CO_2_ in a natural circulation loop.

## Data Availability

The datasets generated and/or analyzed during the current study are available with the corresponding author on reasonable request.
