# Supplementary material for: Heat transfer enhancement using CO2 in a natural circulation loop
Source: Sci Rep. 2020 Jan 30;10:1507. doi: 10.1038/s41598-020-58432-6 (PMC6992701; doi:10.1038/s41598-020-58432-6)
Supplement: Supplementary file 1 — Heat transfer enhancement using CO2 in a natural circulation loop. [file 41598_2020_58432_MOESM1_ESM.docx]

**Heat transfer enhancement using CO_2_ in a natural circulation loop**

Thippeswamy L. R.^1^, Ajay Kumar Yadav^1, *^

^1^National Institute of Technology Karnataka, Surathkal, Mangalore -575025, India. ^*^Corresponding author, email: [ajaykyadav@nitk.edu.in](mailto:ajaykyadav@nitk.edu.in); ajayyadav.aba@rediffmail.com.

**Supplementary files related to experimental setup details**

| 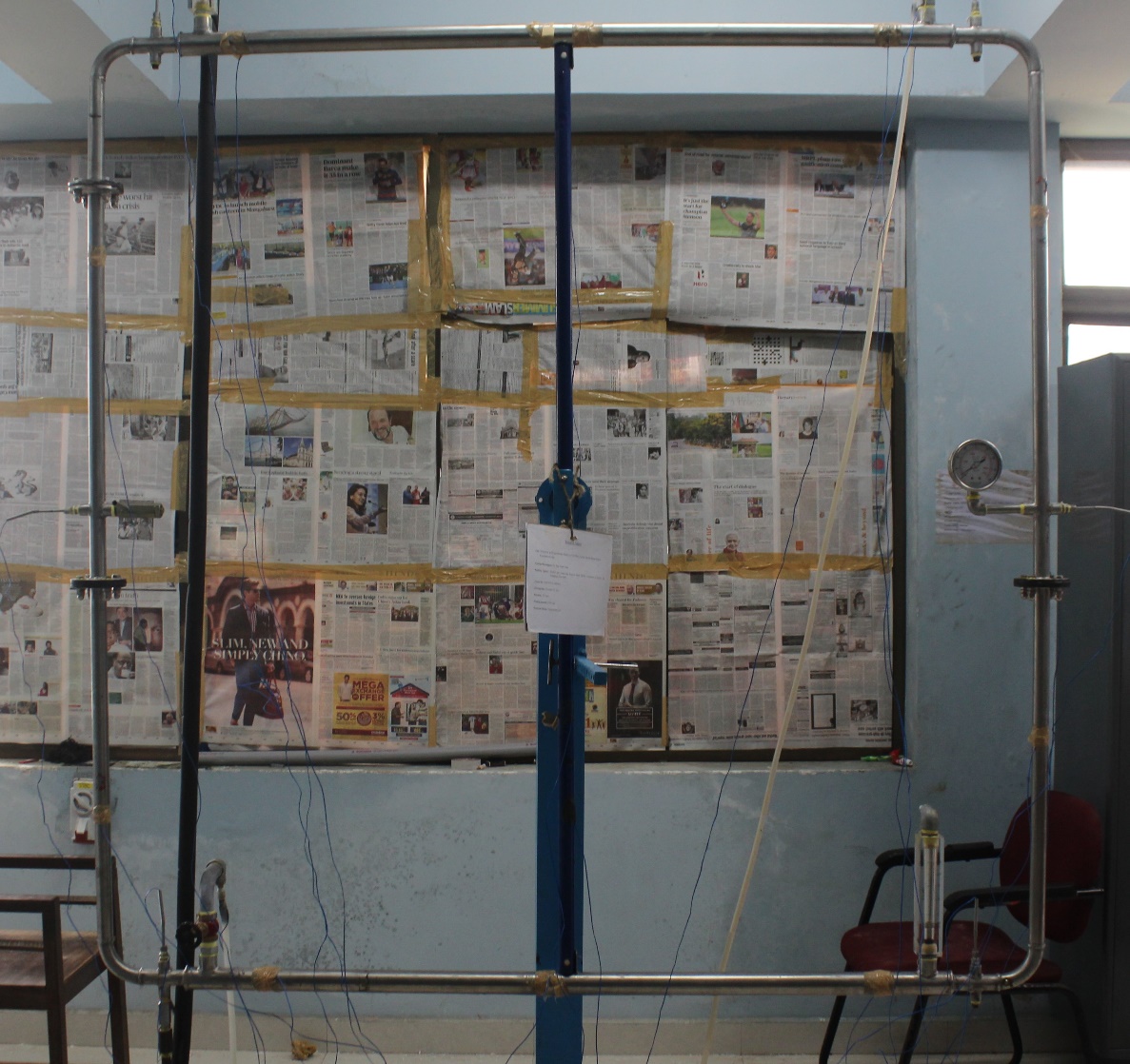 |
| --- |
| **Figure. 1 Experimental setup photographic view without insulation** |

| 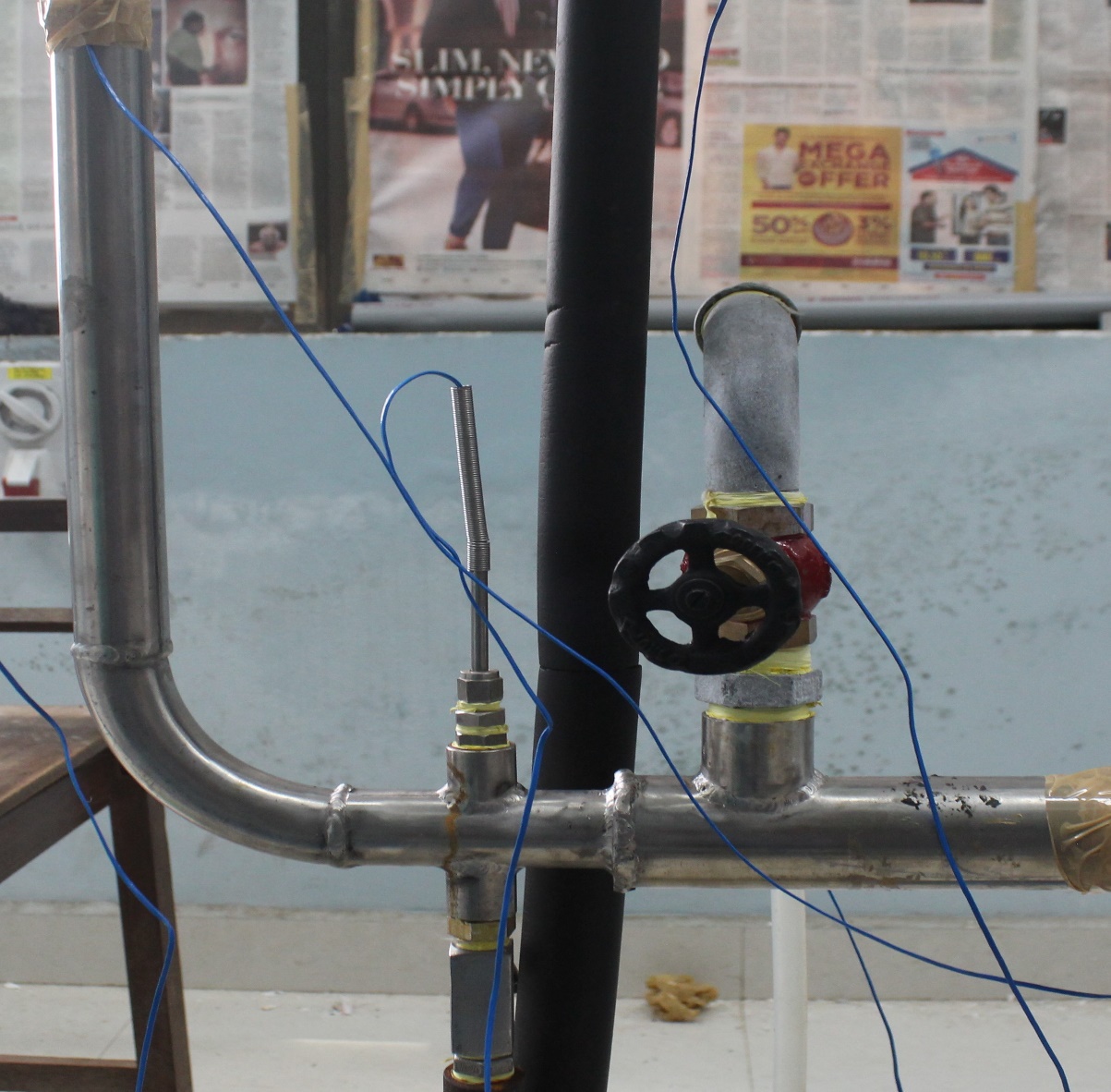 |
| --- |
| **Figure. 2 Photographic view of CO_2_chaging inlet, heat exchanger and thermocouple connection** |

| 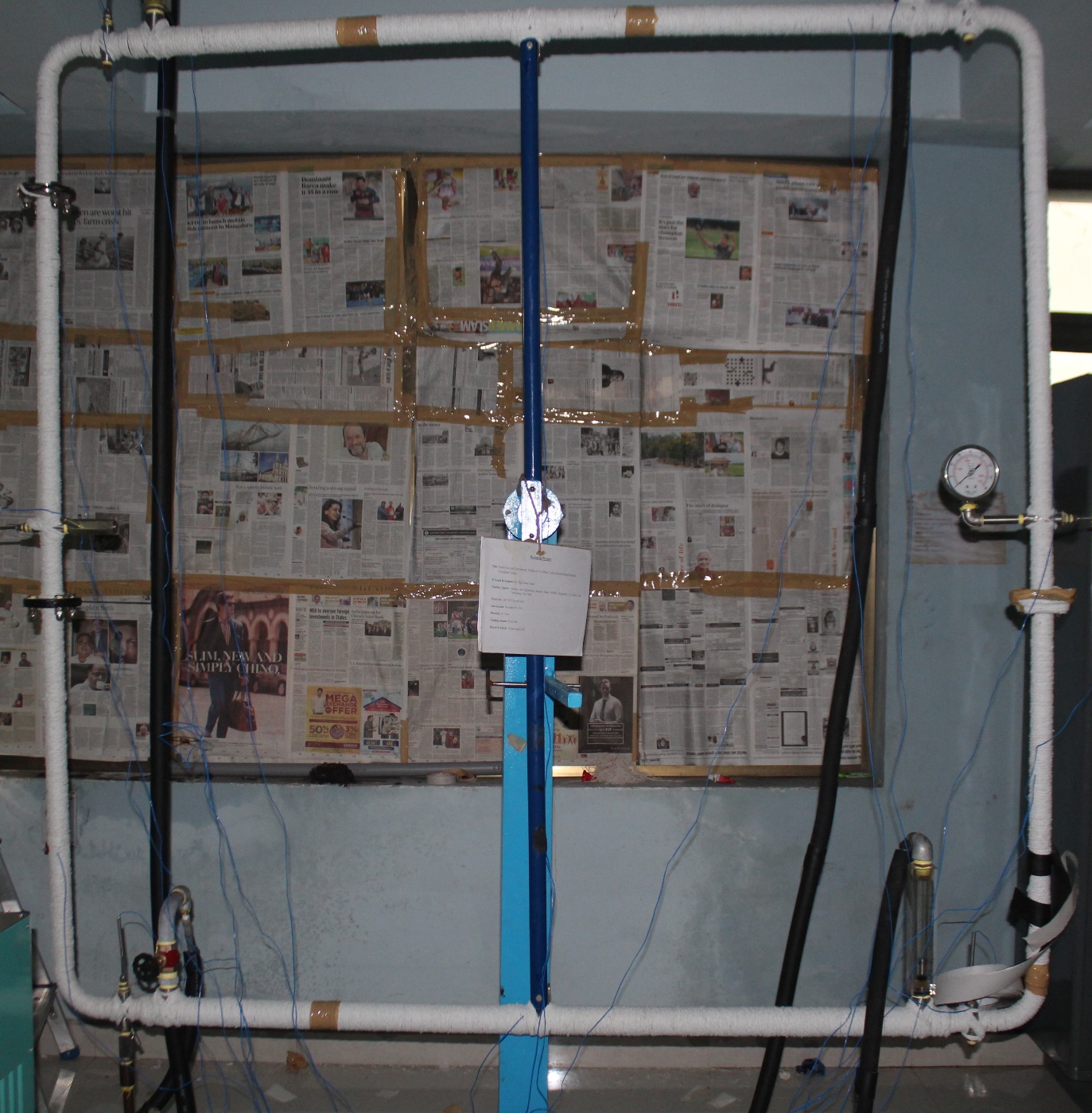 |
| --- |
| **Figure. 3 Experimental setup photographic view without insulation** |

**Various measuring instruments used in the experiments are listed below:**

**Specifications and other details are given below:**

**Data acquisition system:**

Make : KEITHLEY

Model : 2700

Input channel capacity : 40

Least count : ±0.01oC

Temperature Sensors:

Type : T-type

**Thermostatic Bath**

*Hot thermostatic bath*

Make : Thermo scientific

Model : PC200

Range : -60oC to 200oC

Accuracy : ±0.01oC

Pump capacity(max pressure) : 5 bar

*Cold thermostatic bath*

Make : Thermo scientific

Model : PC200

Range : -60oC to +200oC

Accuracy : ± 0.1oC

Pump capacity(max pressure) : 5 bar

**Rotameter**

Range : 0-25 LPM

Least count : 0.25 LPM

**Pressure measuring devices**

*Pressure gauges*

Make : Delta

Range : 0-150 bar

Least count : 0.5bar

*Differential pressure transducer*

Make : Honeywell

Range : 0-10000 Pa

Least count : ±10Pa

**Safety valve**

Make : Swagelok

Range : 51.5- 105 bar
